# Cortical Plasticity Induced by Spike-Triggered Microstimulation in Primate Somatosensory Cortex

**DOI:** 10.1371/journal.pone.0057453

**Published:** 2013-03-05

**Authors:** Weiguo Song, Cliff C. Kerr, William W. Lytton, Joseph T. Francis

**Affiliations:** 1 Departments of Physiology and Pharmacology, SUNY Downstate Medical Center, Brooklyn, New York, United States of America; 2 School of Physics, University of Sydney, Sydney, New South Wales, Australia; 3 Department of Neurology, Kings County Hospital, Brooklyn, New York, United States of America; 4 The Robert Furchgott Center for Neural and Behavioral Science, SUNY Downstate Medical Center, Brooklyn, New York, United States of America; 5 Joint Graduate Program in Biomedical Engineering SUNY Downstate and NYU-POLY, Brooklyn, New York, United States of America; McGill University, Canada

## Abstract

Electrical stimulation of the nervous system for therapeutic purposes, such as deep brain stimulation in the treatment of Parkinson’s disease, has been used for decades. Recently, increased attention has focused on using microstimulation to restore functions as diverse as somatosensation and memory. However, how microstimulation changes the neural substrate is still not fully understood. Microstimulation may cause cortical changes that could either compete with or complement natural neural processes, and could result in neuroplastic changes rendering the region dysfunctional or even epileptic. As part of our efforts to produce neuroprosthetic devices and to further study the effects of microstimulation on the cortex, we stimulated and recorded from microelectrode arrays in the hand area of the primary somatosensory cortex (area 1) in two awake macaque monkeys. We applied a simple neuroprosthetic microstimulation protocol to a pair of electrodes in the area 1 array, using either random pulses or pulses time-locked to the recorded spiking activity of a reference neuron. This setup was replicated using a computer model of the thalamocortical system, which consisted of 1980 spiking neurons distributed among six cortical layers and two thalamic nuclei. Experimentally, we found that spike-triggered microstimulation induced cortical plasticity, as shown by increased unit-pair mutual information, while random microstimulation did not. In addition, there was an increased response to touch following spike-triggered microstimulation, along with decreased neural variability. The computer model successfully reproduced both qualitative and quantitative aspects of the experimental findings. The physiological findings of this study suggest that even simple microstimulation protocols can be used to increase somatosensory information flow.

## Introduction

Electrical neural stimulation has been used in a wide variety of clinical and experimental applications [1]–[4]. Microstimulation in primary somatosensory cortex (S1) has been used to deliver behaviorally relevant information to both humans [5] and animals [1], [6]–[13]. Thus, sensations lost due to disease, injury, or trauma could be restored by microstimulation in primary somatosensory cortex [6], [7], [9], [12], [14] or thalamus [15]–[19]. For example, Leal-Campanario et al. [20] showed that rabbits were unable to distinguish between physical whisker stimulation and electrical stimulation of the vibrissae area of primary sensory cortex. Sensory feedback thus achieved could be used in conjunction with neurally controlled prosthetic limbs properly fitted with joint and touch sensors. However, microstimulation may cause cortical changes that could compete with natural neural processing or potentiation [8], [10], [21]–[26]. For example, microstimulation has recently been shown to “hijack” natural motor intent [27].

Closed-loop and time-dependent microstimulation have been used in deep brain stimulation [28], sensory neuroprosthetics [29], and for treatment of neural disorders such as epilepsy [30]. It is well-known that long-term potentiation (LTP) and long-term depression (LTD) can be induced by Hebbian-type paired microstimulation or high/low frequency microstimulation [31], [32]. The association between LTP and learning has been known for some time [33]; for example, excessive LTP may “saturate” synapses and thus prevent future learning [34], suggesting that behavioral improvements following microstimulation may be related to a balancing of LTP and LTD. In addition, spike-timing-dependent plasticity has been observed in acute slice recordings [35], *in vivo* preparations [31], [36], and in behaving animals [37]; this might promote synchronized firing among cells [38]. Cells in motor cortex used to control an actuator in closed-loop BMIs demonstrate plasticity and reorganization [39]–[41], and functional connectivity changes have also been observed after time-dependent stimulation [21], [37]. However, little is known about how microstimulation in S1 may modify neural activity, how different microstimulation protocols may affect neural responses in S1, and how such changes influence neural information processing.

Our aim was to address these questions by exploring the effect of microstimulation on network reorganization and sensory information encoding of S1. We hypothesized that spike-triggered microstimulation should lead to Hebbian plasticity if the activity at the recording and stimulating electrodes is synchronous. To test this hypothesis, we implanted multi-electrode arrays in the hand representation region of S1 (Brodmann area 1), and analyzed the neural responses evoked by natural touch before and after microstimulation. Both spike-triggered and random stimulus sequences were used. We tested for changes in neural firing rates, variability, and changes in neural synchrony, as measured by unit-pair mutual information and multi-information. To avoid confounds associated with motivation and reward that can affect cortical plasticity, we conducted this microstimulation in quiet, awake animals.

While microstimulation can be a highly informative experimental technique, it has two major drawbacks. First, the number of neurons that can be reliably recorded from and stimulated in a given experiment is small: typically fewer than 100. Second, since the amplitude, polarity, and duration of the stimulation can be independently controlled at each electrode, the parameter space of possible stimulation protocols is extremely large. It is not feasible to explore this space thoroughly with *in vivo* experimentation. To overcome these limitations, we developed a computer model of microstimulation in the thalamocortical system, based on a recently developed spiking-neuron network model [42]. We briefly introduce and present results from this model, and show that these results are broadly consistent with the *in vivo* findings. In future, we will explore the possibility of using this model as a supplement to experimental investigations of microstimulation, particularly with respect to the development of a somatosensory neuroprosthesis [17], [19], [43].

## Materials and Methods

### Animal Welfare

This study was performed in accordance with the recommendations in the Guide for the Care and Use of Laboratory Animals of the National Institutes of Health. The protocol was approved by the Institutional Animal Care and Use Committee of SUNY Downstate Medical Center.

Overall care was managed by the Division of Laboratory Animal Resources (DLAR). Subjects were looked after daily by the senior DLAR staff. The in-house veterinary doctor checked the subjects before they were put on the study, and performed blood tests and physical examinations as needed. Subjects were given weekly fruit or dry treats as a means of enrichment and novelty. There were daily observations of the subject’s body weight, urine color, mucosa and skin turgor, awakeness and alertness, and overall behavior; any abnormal changes were immediately reported to a veterinary doctor. The headpost was used for head restraint 6 weeks after implantation. The subjects resumed normal behavior in the cage a few days after headpost implantation. Microelectrode implantation and connection of headstages with the array connectors did not result in any permanent changes in the subjects’ behavior. In collaboration with DLAR, we have attempted to offer as humane treatment of our subjects as possible, and we believe that the standard of animal care and welfare in our lab exceeds national guidelines.

### Surgical Procedure

Two macaques (JK: male, 3.9 kg; AC8: female, 4.1 kg) were used in these experiments. These animals were primarily trained for and used in other studies on brain-machine interface development that will be published separately. Two types of surgeries were performed on the subjects: headpost mounting surgery and microelectrode array implant surgery. Surgeries were performed in a dedicated operating room suite in an aseptic manner. Anesthesia and initial preparations of the surgery were done by the in-house veterinary doctor personally or under her direct supervision. Isofluorane anesthesia was used throughout the surgery. Injectable steroids were used to minimize brain edema and swelling and mannitol and diuretics like furosemide were kept on side if needed. Surgeries were performed by researchers with formal array implantation training and/or human surgical experience. Subjects were given appropriate analgesics, antibiotics and other needed medications by injection throughout the course of surgery and in the post-operative convalescence (commonly 2 weeks). The subjects were observed hourly or 2-hourly for the first 12 hours after surgery by the lab personnel and were inspected daily or twice daily by the DLAR personnel.

**Figure 1:**
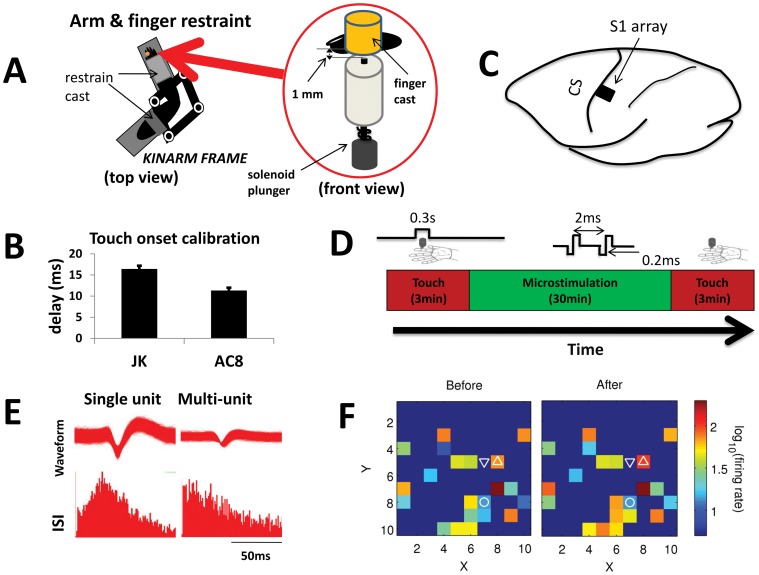
Training protocols and general observations. (**A**) Monkeys were seated comfortably in a non-human primate chair with their right arm and hand restrained in a KINARM. The finger to undergo tactile stimulation was fixed in a finger cast (customized based on individual finger and monkey) just above the solenoid plunger. (**B**) The delay from the solenoid control pulse to the plunger touching the skin was calibrated with a force sensor (error bars show SD). (**C**) Multi-electrode arrays were implanted close to the central sulcus (CS), in the hand representation area (S1 area 1). (**D**) Daily training sessions were comprised of three segments: tactile stimulation, microstimulation, and tactile stimulation. Each tactile stimulus had a duration of 0.3 s and indented the skin by 1 mm, and was applied with a mean frequency of 0.5 Hz. Microstimulation consisted of two biphasic pulses of amplitude 50 µA, and were triggered by spikes recorded from the reference unit, subject to a 5 ms delay. (**E**) Typical waveforms and inter-spike intervals (ISIs) of single and multi-units. (**F**) Activation maps show firing rate as a function of electrode position; each square corresponds to one electrode in the 10×10 array. There were broad cortical responses to tactile stimulation. The stimulating source is shown by the upward triangle, the stimulating sink by the downward triangle, and the triggering reference unit by the circle.

Head-posts were implanted at least 6 weeks before microelectrode array implantation. The detailed procedure for head-post and cortical implantations has been described previously [44]. Briefly, monkeys were initially anesthetized by using ketamine (20 mg/kg) followed by intubation and ventilation with isoflurane gas (0.75–2%) and oxygen, then a midline incision was made and the skin was retracted to expose the skull. Two 20 mm by 20 mm bone flaps were removed on each side of the midline over the S1/M1 hand region. After a dura flap was cut and open, four 100-electrode platinum-iridium arrays (Blackrock Microsystems) were pneumatically inserted in the hand representation area of both the primary motor cortex (M1) and S1 bilaterally. The S1 implant was placed as close as possible to the central sulcus on the postcentral gyrus (as shown in Figure 1C), to ensure that we were recording from area 1. We only present the data from the left S1 (contralateral right hand) in this report. The somatotopy in S1 was tested by touching and manipulating the hand and fingers; however, we did not specifically identify purely cutaneous or proprioceptive units for this work, and instead concentrated on units that responded to our tactile stimulator. Following the surgery, analgesics (Buprenex, 0.02 mg/kg; RimadyI, 2 mg/kg) and antibiotics (Baytril, 6 mg/kg) were administered to the recovering animals for 3 and 5 days, respectively. Bicillin (1 ml/20 lbs) was administered every other day for 14 days. Recording began at least three weeks after cortical implantation.

### Neural Recording and Offline Sorting

Neural data were recorded with multichannel acquisition processors (MAP, Plexon Inc.). Unity-gain preamplifier head-stages (Plexon Inc.) were connected to the ICS-96 array connectors (Blackrock Microsystems) via flexible wires. Neural signals were band-pass filtered (154 Hz to 8.8 kHz) and digitized at a sampling frequency of 40 kHz. Spikes were detected online when they passed a threshold level (roughly 3 standard deviations [SD] above background level), which was manually set and then kept unchanged throughout the day. The detected spikes were automatically classified online by using waveform sorting methods based on either principal component analysis or template matching (Plexon Inc.). The detected spiking times and spike waveforms were saved for offline analysis. Units (single units and multi-units) were isolated offline using the Offline Sorter (Plexon Inc.). Single units were defined if there were clear waveform shapes, well-separated in a feature space, with interspike intervals greater than 1.6 ms. We took poorly isolated units to be multi-units (see Figure 1E); however, we did not differentiate between single and multi-units for the purposes of this work. Up to 50 simultaneous channels of usable neural activity were recorded from the array, and one or two individual units from several of the channels could be isolated.

### Stimulation Protocols

The stimulation system and protocol are shown in Figure 1. Subjects were seated comfortably in a non-human primate chair (BKIN Technologies) in a sound-proof recording room with their right arms and hands restrained to the KINARM (BKIN Technologies). The finger to undergo tactile stimulation was fixed in a finger cast (which was customized based on the individual finger and subject) just above the solenoid plunger (Figure 1A). The monkeys were trained to be seated in the chair quietly; they did not perform any behavioral task. Each daily training session was composed of three phases (Figure 1D): 3 min of tactile stimulation, 30 min of microstimulation (either spike-triggered or random), then 3 min of tactile stimulation. Ten stimulation sites in the two subjects (four in JK and six in AC8) were recorded from during 15 different sessions.

### Tactile Stimulator

Tactile stimulation was produced using a push-type solenoid actuator (STA-195201, Ladex Inc.), which was mounted under a finger cast (Figure 1A). The finger and hand casts were attached to an exoskeletal robotic system KINARM (BKIN Technologies). The KINARM was locked in place during tactile stimulation to keep the arm in the same configuration across sessions. The solenoid was controlled by a personal computer and digital card (PCI-6229, National Instruments Inc.) using a customized program written in MATLAB (The MathWorks Inc.). A return spring was used to pull the plunger back after each stroke. The diameter of the solenoid plunger, which indents the skin, was 1 mm. The tactile stimuli could be applied to different digits. The system latency from tactile stimulation control pulse to the time of solenoid plunger touching skin was calibrated with a force sensor (Figure 1B). Each tactile stimulation control pulse produced skin displacement around 1 mm for 0.3 s, and the tactile stimulus was applied randomly with a mean frequency of 0.5 Hz, as shown in Figure 1D. Peristimulus intervals were calculated relative to skin touch time.

### Electrical Microstimulation Protocol

Spike-triggered microstimulation was based on a reference unit recorded from the S1 hand region, since this microstimulation paradigm has previously been shown to induce plasticity [22], [35], [37], [45]. These triggering units (which could be either single- or multi-units) had an average spontaneous firing rate of ∼10 Hz, and hence roughly 18000 spike-triggered microstimulation pulses were applied in each 30 min session. No tactile stimulation was applied during the microstimulation period. In addition, we tested random microstimulation, with the mean and standard deviation of this random input set to match the statistical properties of previously recorded spike-triggered sequences. As a further control, we used previously recorded spike recordings in subsequent sessions; there were no obvious differences between these and the random input.

Microstimulation pulses were delivered a short delay (5 ms) after each spike recorded from the reference channel (conditioning unit) on the S1 array. A delay of 5 ms was used since this latency has previously been shown to induce potentiation [37]. The conditioning unit was chosen randomly from the subset of touch-responsive units, but was not specifically classified as proprioceptive or cutaneous. The spike timing of the conditioning unit was taken from the MAP system (Plexon Inc.) and used to control a digital IO card (PCI-6229, National Instruments Inc.) to trigger the microstimulation. The microstimulation was delivered via an isostimulator (Model 2200, A-M Systems Inc.) to a bipolar pair of adjacent electrodes taken at random from the electrode array. The stimulating electrodes were chosen to be an adjacent pair to reduce current spread. The real-time control program for the digital card was written in C (LabWindows/CVI, National Instruments Inc.) and ran on a personal computer. It was capable of producing any arbitrary stimulation pattern (including variables such as pulse width, amplitude, and frequency). The stimulation parameters were chosen based on previous reports [21], [37] and our preliminary results [46]. Thus, cathode-leading biphasic double-pulse stimuli were used for the stimulation pulse, as shown in Figure 1D. These pulses had an amplitude of 50 µA, pulse width of 200 µs per phase, and a pulse interval of 2 ms. This pulse sequence was motivated by its similarity to the high-frequency doublets often seen in S1 in response to natural touch [47].

### Data Analysis

#### Firing rate modulation

**Figure 2:**
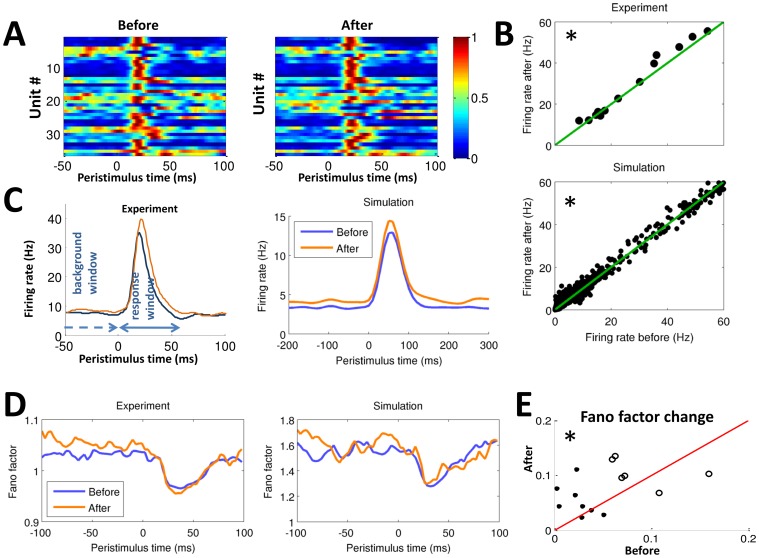
Ensemble (network level) responses and dynamics before and after microstimulation. (**A**) Example response patterns, showing normalized firing rate as a function of peristimulus time and unit number. (**B**) Ensemble firing rates increase following microstimulation in both experiment (session averages shown) and simulation; however, the simulation provides access to 100 times as many units. (**C**) Peristimulus time histograms. Similar response shapes are seen in the experiment and simulation, including increased prestimulus activity and increased peak firing rate. (**D**) Changes in the Fano factor averaged across all units. Neural variability decreases during tactile response, an effect enhanced following microstimulation in both the experimental and simulated conditions. (**E**) Stimulus-induced reductions in the Fano factor before and after experimental microstimulation across sessions. A larger post-stimulus decrease in the Fano factor is seen following microstimulation. Circles and dots show data from subjects JK and AC8, respectively. * = *p*<0.05.

In order to test the effect of microstimulation on firing rate changes during tactile stimulation, peristimulus time histograms (PSTHs) were constructed for the tactile stimulation trials both before and after microstimulation. The PSTHs were smoothed with a Gaussian density kernel with a 20 ms window to obtain the firing rate (Figure 2C). Then, the peak firing rate and the area under the response curve were calculated from the PSTHs for the 0–70 ms poststimulus time window, with baseline defined by the time window starting 250 ms before the stimulus and up to but not including 0 ms prestimulus time. These windows were chosen based on the observed neural responses to touch stimulation. If the peak firing rate in the response window was above 3 SD of the baseline firing rate, the cell was classified as touch responsive; otherwise it was classified as nonresponsive. Neural response sharpness was defined as the ratio of the peak firing rate to the temporal width of the response curve at a height of 3 SD above baseline. We did not analyze the off responses (neural responses due to the retraction of tactile stimulation).

#### Fano factor analysis

The Fano factor characterizes the variability of neural activity, with a Fano factor of one corresponding to a purely Poisson process. The firing rate and the Fano factor, when combined, provide estimates of the dynamics of neural firing [48]. The Fano factor is defined as
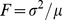
where 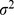 and 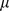 are the variance and mean of spike counts within a given 20 ms window. These windows were then shifted in 3 ms increments to produce Figure 2D.

#### Unit pair mutual information and multi-information

To determine how much information was encoded or shared between units before and after microstimulation, we calculated the mutual information for pairs and small ensembles of cells. Mutual information has been successfully used for analyzing statistical dependencies between spiking neural systems [49]. Unlike the traditional linear cross-correlation method, mutual information tests for both linear and nonlinear dependences between pairs of units. In order to estimate the mutual information between unit pairs, spike trains were binned in 10 ms windows, which resulted in values of either zero or one for most bins. Shorter and longer time bins were tested, and gave similar results with respect to changes observed before vs. after microstimulation. The absolute value of the mutual information increased with bin size, as did the statistical power of the findings, but a relatively short bin size was used to reduce the potential for bias. The mutual information of unit pairs was calculated as
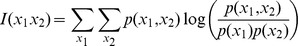
where 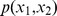 is the joint probability distribution function of 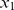 and 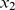, which are spike counts of a pair of units, with 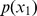 and 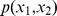 being the marginal probability distribution functions of units 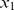 and 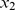, respectively.

Parallel to the unit-pair mutual information, the multi-information was used as a measure of the total ensemble, as an alternative to looking at all possible mutual information unit pairs. The multi-information expresses the amount of redundancy or dependency existing among a set of variables. An increase in multi-information indicates that there is an increase in information shared between the ensemble units and thus a higher dependency among its members. The multi-information of the ensemble units was calculated as
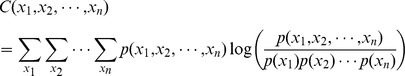
where 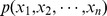 is the joint probability distribution function of 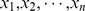, and 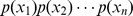 are the marginal probability distribution functions of 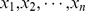, respectively.

Information calculated by using the empirical probability distribution will most likely have a bias when the number of samples is limited, but this bias problem can be alleviated by the use of bias correction techniques. When the number of trials is large enough that every possible response occurs many times, analytical approximations to the bias can be estimated [50]. In the absence of knowledge about specific features of the neural response statistics that favor one specific procedure over another, we estimated the mutual information by using a shuffling bias reduction procedure. Thus the estimated information was bias-corrected after subtracting the shuffled information, as implemented in an information-breakdown toolkit [51].

#### Statistical analysis

In order to evaluate the effect of microstimulation on the network, we defined the percentage change *PC* as
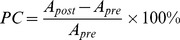
where 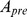 and 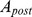 were parameters before and after microstimulation, respectively.

For the ensemble or network-level analysis, we found there were some differences between the two subjects, such as one subject having a higher firing rate and unit-pair correlation than the other. However, the effects of microstimulation on the percentage changes were similar, with no statistically significant differences between the subjects, so we pooled the data from the two subjects by considering each session as an observation for consideration in our statistical testing. Then the following statistics were used: (1) non-parametric Wilcoxon signed-rank tests were used to test whether the percentage changes (of firing rates, Fano factor, mean mutual information, and multi-information) were significantly changed after microstimulation; and (2) two-sample Kolmogorov-Smirnov tests were performed to compare whether pre- and post-microstimulation condition variables were likely to have come from the same distribution or not. All significance levels of the above tests were set at 0.05, unless stated otherwise. All data analyses were performed using MATLAB (The MathWorks Inc.).

### Computer Model

The computer model used here was based closely on Kerr et al. [42], following earlier work by Lytton, Neymotin, and others [52]–[55]. This model of the thalamocortical system uses leaky integrate-and-fire neurons. These neurons are capable of capturing much of the phenomenology of real neurons (such as bursting, adaptation, and depolarization blockade), while still being simple enough to connect into large networks. The dynamics of each neuron is a function of a single state variable representing the membrane voltage. This voltage is updated based on one of three events:

1. Synaptic input: 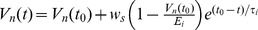, where 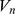 is the membrane voltage of neuron 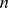; 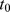 is the synaptic event time (i.e., 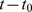 is the time since the event); 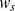 is the weight of synaptic connection *s*; 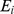 is the reversal potential of ion channel *i*, relative to resting membrane potential (where *i* = AMPA, NMDA, or GABA_A_, and 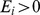 for depolarizing inputs and 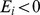 for hyperpolarizing); and 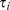 is the receptor time constant. Synaptic weight 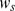 is incremented via spike-timing-dependent plasticity according to 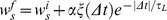, where 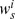 and 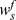 are the initial and final weights of the synapse, respectively; 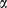 is the plasticity increment (equivalent to a learning rate); 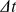 is the temporal difference between synaptic input and neuronal firing; 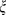 is a sign function that specifies potentiation or depression via 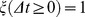 and 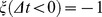; and 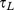 is the learning window (10 ms). Maximal synaptic weights were 5 times the synaptic weight at baseline, in line with experimentally observed results [35].
2. Action potential: a neuron fires an action potential at time *t* if 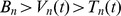, where 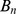, 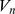, and 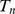 are the blockade voltage, membrane voltage, and threshold voltage, respectively, for neuron *n*. Action potentials arrive at target neurons at time 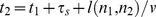, where 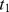 is the time the first neuron fired, 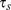 is the delay due to synaptic conduction effects, 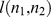 is the axon length between neurons 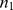 and 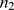, and *v* is the axonal conduction velocity.
3. Refractory period: after firing, a neuron cannot fire during the absolute refractory period, 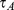. Firing is reduced during the relative refractory period by two effects: first, an increase in threshold potential, 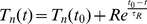, where *R* is the relative refractory change in threshold voltage and 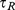 is its time constant; and second, by hyperpolarization, 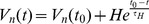, where *H* is the amount of hyperpolarization and 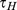 is its time constant.

These neurons were connected together to form thalamocortical networks using empirical data on intrathalamic, thalamocortical, interlaminar, and intralaminar connection densities. In total, the model consisted of 1980 neurons distributed among 14 neuronal populations (inhibitory thalamic reticular neurons; excitatory thalamic relay neurons; and excitatory pyramidal neurons, fast-spiking interneurons, and low-threshold-spiking interneurons for cortical layers 2/3, 4, 5, and 6) in accordance with empirical data on relative numbers of neurons in each layer. The number of neurons in the model is constrained to be integer multiples of 495 by the relative number of neurons in each population. Models consisting of 495 to 9900 ( = 20×495) neurons were tested. The model with 1980 ( = 4×495) neurons produced very similar results to larger models, so this model was used to minimize computation time. Excitatory neurons differed from inhibitory ones in terms of the parameters used in the equations above, while populations in different layers were defined by having different connectivities. Tables of parameter values and connectivities are provided in Neymotin et al. [54]. Connections between neurons within a single layer were spatially dependent, with a given connection having probability 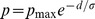, where 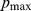 is the maximum connection probability; 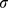 is the rate of fall-off of connection probabillity; and *d* is the distance between the two neurons, defined as 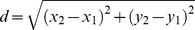, where 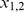 and 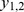 are the spatial coordinates of the two neurons. Values used for 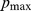 and 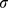 were based on empirical data.

Natural touch stimuli were modeled as suprathreshold inputs to the 25% of the thalamocortical relay cells that were closest to the center of the model (representing a spatially contiguous field induced via point stimulation of the periphery); the precise fraction of stimulated cells was not found to significantly affect the results of the simulation. Touch stimuli were delivered with a mean period of 600 ms (range: 400–800 ms). As in the experiment, microstimulation was either triggered by the firing of a randomly-chosen cell, or applied randomly with the same frequency and variance as in the spike-triggered case. In both cases, microstimulation was applied to 10% of all pyramidal neurons in cortical layer 5; in contrast to natural touch stimuli, these cells were chosen randomly rather than contiguously, since microstimulation is thought to activate axons, rather than cell bodies, close to the electrode [56]. Each simulation lasted for 300 s of simulated time, and was comprised of 100 s of tactile stimulation; 100 s of random microstimulation, spike-triggered microstimulation, or tactile stimulation; and then 100 s of tactile stimulation.

The model was implemented in NEURON 7.2 [57], [58] for Linux, and is available on ModelDB (https://senselab.med.yale.edu/ModelDB/ShowModel.asp?model=147579). Each 300 s simulation took approximately 40 min to run on a single Intel Xeon 2.3 GHz core.

## Results

We recorded simultaneous neural activity in the somatosensory cortex area 1 (Figure 1C) from 15 sessions in the two subjects (JK, N = 8; AC8, N = 7). Single and multi-units were used for our analysis without differentiation. Subject JK showed more multi-unit activity than AC8; while this affected absolute values of firing rate and mutual information, we did not observe significant differences with respect to microstimulation-induced changes, and thus data from the two subjects were analyzed together. An average of 42±8 (mean±SD) units were recorded from in each session. Electrical stimulation was delivered at 10 sites between the two subjects (JK, N = 4; AC8, N = 6). To determine the influence that microstimulation had on the processing of natural tactile stimulation, we analyzed the firing characteristics of the ensemble during tactile stimulation trials both before and after the microstimulation period. No microstimulation was given during periods of touch stimulation, nor were touch stimuli applied during microstimulation. Following careful removal of the stimulation artifact, the conditioning phases during microstimulation were analyzed in 12 sessions.

### Changes in Ensemble Firing Rate

The ensemble responses were analyzed for peak firing rate, sharpness of the response curve, and area under the response curve. Tactile stimulation resulted in widespread neural responses, with a similar but stronger neural response observed after microstimulation. Units simultaneously recorded in S1 displayed a distribution of different response amplitudes (Figure 1F) and latencies (Figure 2A) to tactile stimulation at the finger. At the population level, the ensemble showed significant tactile responses (defined as >3 SD above baseline firing rate) in all the recording sessions, as would be expected for this cortical region (Figure 2C). Following microstimulation, statistically significant increases were seen in the peak firing rate (8%), area under the curve (11%), and response sharpness (15%), with all changes being statistically significant (Wilcoxon signed-rank test, *p*<0.05). Similar results were obtained in the model, which also showed an increased average firing rate following microstimulation (Figure 2B) of comparable magnitude (15±2%). The model also found similar changes in the area under the curve (16±3%) and response sharpness (34±6%), as shown in Figure 2C. In short, these findings indicate that spike-triggered microstimulation increases the population response in area 1 to tactile stimulation and that such changes are in agreement with spike time dependent plasticity rules. Note that many of the quantitative differences between experimental and model results (for example, baseline firing rates of 10 Hz and 3 Hz, respectively) can be accounted for by the fact that the experimental results include multi-units as well as single units.

### Changes in Ensemble Variability

The ratio of the variance and the mean of the firing rate, called the Fano factor, is a common characterization of neural dynamics [59], since changes in variability are often seen with changes in response amplitude. If spikes are generated by a perfect Poisson process, the Fano factor will be unity; the importance of Poisson processes as models of neural activity explain why the Fano factor is more commonly used in neuroscience than equivalent measures, such as the dimensionless coefficient of variation (defined as the ratio of the standard deviation to mean).

Before microstimulation, the Fano factor decreased by an average of 7% following tactile stimulation, as shown in Figure 2D. After microstimulation, the decrease was 9%, as shown in Figure 2E, although some of this enhanced decrease is attributable to the fact that the prestimulus Fano factor was higher after microstimulation. This indicates that the neural response to touch was more stereotyped and less variable between trials after microstimulation compared to before. The decrease in the Fano factor was also observed in individual units across trials (data not shown). Qualitatively similar results were observed in the simulation (Figure 2D), which found pre- and post-microstimulation decreases of 18% and 20%, respectively. These results suggest that spike-triggered microstimulation could potentially be used to decrease the variability of somatosensory information transmission in S1, at least at a neurophysiological level. However, psychophysical testing would be required to determine whether or not these changes are above the threshold of perceptibility.

### Changes in Mutual Information

In order to test how sensory information is encoded between units and how microstimulation affects this encoding, we used several information-theoretic measures. Information theory has been widely used to quantify how much information a neural response carries about a stimulus [49]. Mutual information is a measure of statistical dependence between two variables of interest, such as the stimulus and spike trains [60]. Here we used mutual information as a measure of the dependence between spike trains from pairs of units (either single or multi-units). In addition, we calculated multi-information, which quantifies the amount of redundancy existing among the ensemble of units, and hence provides a measure of stochastic dependence among variables in a network. Unlike linear cross-correlation, these measures capture both linear and nonlinear dependences between units.

**Figure 3:**
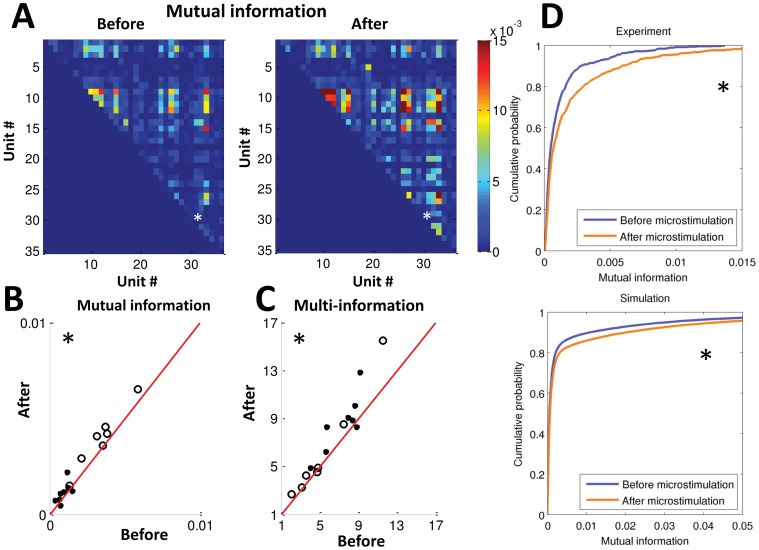
Changes in mutual information (measured in bits). (**A**) Examples of unit-pair mutual information from single sessions before and after microstimulation. The white asterisk indicates the triggering reference unit. (**B**) Mutual information for all sessions, showing a 15% increase following microstimulation. (**C**) Multi-information for all sessions, showing a 25% increase following microstimulation. Circles and dots show data from subjects JK and AC8, respectively. (**D**) Cumulative distribution functions for mutual information in experimental (top) and simulated (bottom) conditions. * = *p*<0.05.

In Figure 3A, we show typical results from unit-pair mutual information analysis over the ensemble recorded in a single session. The unit-pair mutual information was significantly increased following microstimulation, both in terms of individual sessions (Wilcoxon signed-rank test, *p*<0.05, Figure 3B) and across the entire population (Kolmogorov-Smirnov test, *p*<10^−5^, Figure 3D); similar results were observed in the simulation (Figure 3D). The multi-information of the ensemble units also showed a significant increase after microstimulation (Wilcoxon signed-rank test, *p*<0.01), as shown in Figure 3C. Mutual and multi-information increased by 15% and 25%, respectively. The simulation found that mutual information increased by 41% and multi-information by 18%. Despite the relatively small effect size in the experimental result, bootstrapping with 100 samples showed that the increase was statistically significant in 13 out of the 15 sessions. The increased unit-pair mutual information and multi-information suggest an increased functional connectivity in the system following microstimulation. The much greater increase in mutual information found in the simulation is linked to a much higher overall level of mutual information, which is most likely due to the fact that the simulation was able to sample many more adjacent cell pairs, which have higher connectivity and thus higher mutual information.

**Figure 4:**
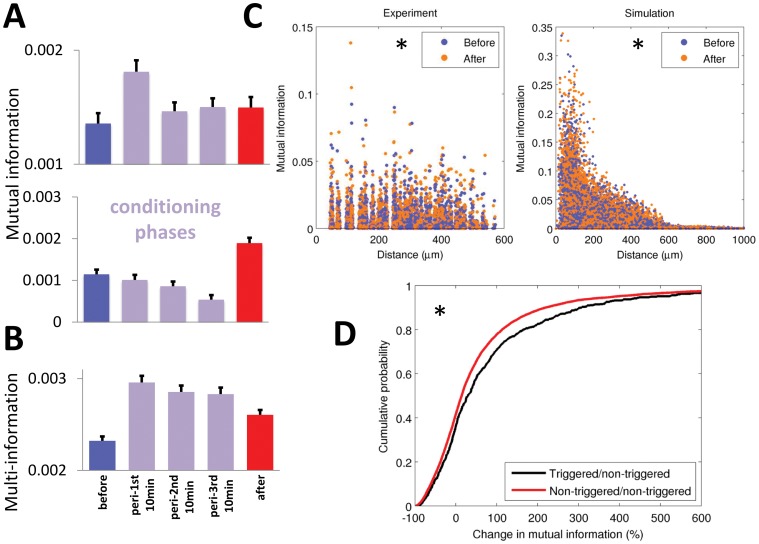
Conditioning effects on mutual information (in bits). (**A**) Two example conditioning phases showing that unit-pair mutual information increased after microstimulation following either intra-stimulation potentiation (top panel) or intra-stimulation depression (bottom panel). (**B**) Conditioning effects for multi-information, showing evidence of potentiation across sessions. (**C**) Mutual information as a function of distance between the units in experimental (left) and simulated (right) conditions. The mutual information was generally higher for nearby pairs than for distant ones, although this effect was less pronounced following microstimulation. In the simulation, the effect was especially pronounced within the first 100 µm. Experimental distances are approximate, since cell body locations cannot be precisely determined from recording electrodes; hence, slight scatter has been added to the electrode separations to show the density of points at each distance. (**D**) The effect of the reference unit used for spike-triggered microstimulation. Unit pairs that included the reference unit showed a greater increase in mutual information following microstimulation than pairs that did not. * = *p*<0.05.

To understand the mechanisms underlying this change in mutual information, the spike-timing dependent conditioning process was analyzed in three consecutive 10 min phases during microstimulation. The observed increases in mutual and multi-information were most likely due to potentiation during microstimulation (Figures 4A and B); this potentiation following microstimulation occurred even in cases where depression occurred during conditioning phases (Figure 4A, lower panel).

It has been reported that neighboring units are more likely to be located in the same functional column, and thus show more highly correlated activity [61]. We tested this by calculating unit-pair mutual information as a function of distance, and found that mutual information was higher for nearby units than for distant ones, as expected; this effect was confirmed by the simulation, which found an especially prominent increase <100 µm, which cannot be easily sampled experimentally (Figure 4C).

We also found that pairs of units that included the spike-triggered reference unit had greater mutual information than pairs that did not (Kolmogorov-Smirnov test, *p*<0.001), as shown in Figure 4D. Overall, 64% triggering unit pairs showed increased mutual information, compared to 58% for non-trigger unit pairs, a statistically significant difference (Wilcoxon rank-sum test, *p*<0.001). This suggests that connections between the triggering unit and its neighbors were more likely to be potentiated than other connections.

**Figure 5:**
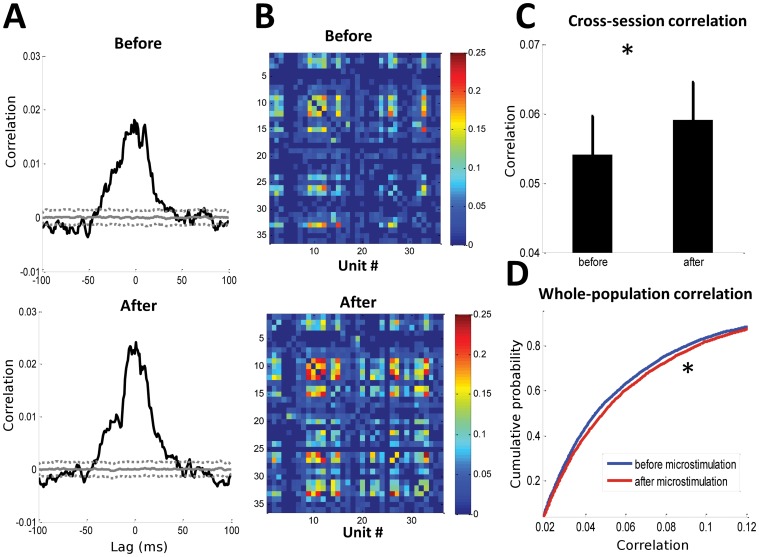
Unit-pair correlation changes. (**A**) Example of a unit-pair crosscorrelogram before and after microstimulation, showing the mean correlation (solid gray line) and 95% confidence interval (dotted gray lines). (**B**) The unit-pair correlation coefficient matrix, constructed from statistically significantly correlated unit-pairs from the ensemble, showed enhanced correlation after microstimulation. The mean correlation coefficient was significantly higher after microstimulation (Wilcoxon sign-rank test, *p*<0.05). (**C**) A statistically significant increase in correlation between units was also observed between units across sessions (Wilcoxon sign-rank test, *p*<0.05). (**D**) The cumulative distribution function for unit-pair correlations before and after microstimulation from the whole population also showed a statistically significant change (Kolmogorov-Smirnov test, *p*<0.05). * = *p*<0.05.

To ensure that these results were not biased by a small number of poorly sampled units, we cross-validated these mutual information findings using a pairwise-based correlation method. This was applied at the level of the individual unit-pair, the network, across sessions, and across the whole population. Examples of unit-pair (Figure 5A) and network (Figure 5B) correlations showed significant increases in correlated firing after microstimulation. There was also a significant increase in the unit-pair correlation when analyzed across either sessions (Figure 5C) or from whole population (Figure 5D). Over the whole population, the correlation of unit pairs increased by an average of 10% after microstimulation. Thus, these results were in agreement with the findings of the information-based analysis.

### Responsiveness to Tactile Stimulation

**Figure 6:**
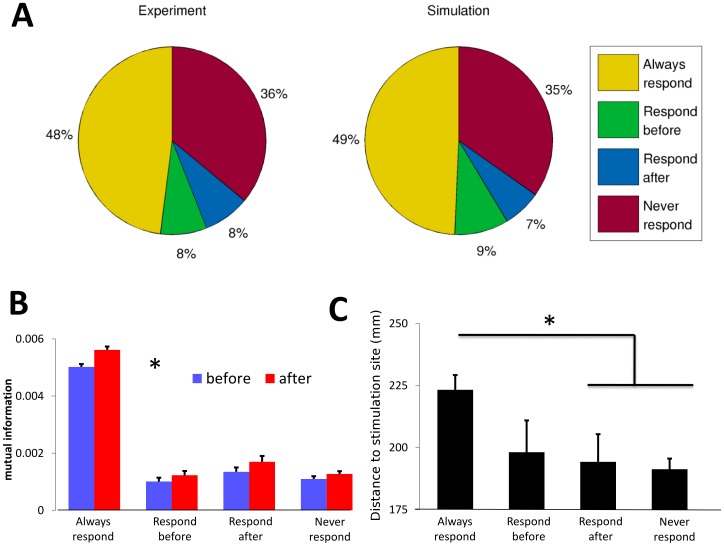
Responsiveness to tactile stimulation. (**A**) We identified four types of units based on whether they responded to tactile stimuli both before and after microstimulation (“Always respond”), responded before but not after microstimulation (“Respond before”), responded after but not before microstimulation (“Respond after”), or did not respond to any tactile stimuli (“Never respond”). Nearly identical proportions of each type were observed in the experiment and in the simulation. (**B**) Mutual information (in bits) as a function of unit responsiveness type. Unit-pair mutual information was significantly increased following microstimulation for each unit type; units that always responded to stimuli had much larger mutual information than other unit types. Error bars show standard error. (**C**) Relationship between unit responsiveness and distance to stimulating electrode. Units that either never responded to tactile stimulation, or only responded before microstimulation, were statistically significantly closer to the stimulating electrode than units that responded both before and after microstimulation. * = *p*<0.05.

We identified four types of units based on whether they responded to tactile stimuli both before and after microstimulation, responded before but not after microstimulation, responded after but not before microstimulation, or did not respond to any tactile stimuli. A plurality of units (48%) responded to tactile stimuli both before and after microstimulation; a small fraction of units lost tactile responsiveness (8%) or acquired it (8%) following microstimulation, while the remainder (36%) did not respond; remarkably, nearly identical proportions as these were observed in the simulation (Figure 6A). Units that responded both before and after microstimulation showed significant increases in peak firing rate, response sharpness, Fano factor, and mutual information (Figure 6B) after microstimulation (Wilcoxon signed-rank test, *p*<0.01 in all cases).

Units that responded both before and after were also located considerably further from the stimulating electrode (Figure 6C). This might result from stimulation-induced depression of neuronal excitability for units close to the stimulating electrode [34], an effect that was clearly observed in the stimulating channels. We also found that the unit-pair mutual information for units responsive only after microstimulation was significantly higher than that of units responsive only before microstimulation (Wilcoxon rank-sum test, *p*<0.01), although their distance to stimulating electrode was not significantly different (Figure 6C). This suggests that spike-triggered microstimulation might have selective effects on different unit types.

### Spike-Triggered vs. Random Microstimulation

**Figure 7:**
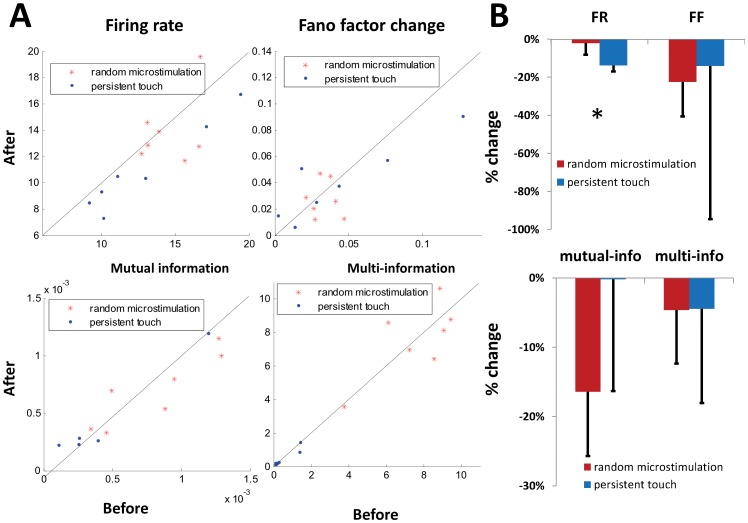
Context-dependent plasticity effects. Different parameters before and after either random microstimulation conditioning or persistent touch conditioning (**A**) and their percentage changes (**B**). Random microstimulation had no statistically significant differences on peak firing rate (FR; in Hz), Fano factor (FF), unit-pair mutual information (in bits), or multi-information (in bits). Persistent tactile stimulation resulted in a statistically significant decrease in firing rate, but no significant changes in other variables. Error bars show standard error. * = *p*<0.05.

In order to test whether the effects described above were a result of spike-triggered microstimulation, we performed additional tests using random microstimulation and persistent tactile stimulation in one of the subjects. During random microstimulation, the frequency of the microstimulation pulse sequence had the same mean and variance as in spike-triggered microstimulation; other stimulation parameters were also kept constant. In contrast to spike-triggered microstimulation, there were no statistically significant differences in the firing rate, unit-pair mutual information, or ensemble multi-information before and after microstimulation (Figure 7A), although all four measures decreased in value following microstimulation. This suggests that random microstimulation protocols have different effects on S1 processing compared with spike-triggered microstimulation. No statistically significant effects were observed between random and spike-triggered microstimulation in the model, however. We attribute this to the fact that the model, unlike the real brain, does not exist in a larger oscillatory environment. Hence, whereas experimental random microstimulation pulses may be in-phase or antiphase with respect to global brain activity (such as the alpha rhythm), this does not apply to the model, which represents too small a region of the brain to support pronounced, large-scale oscillatory behavior.

To test the effect of persistent tactile stimulation, we applied tactile stimuli with a mean frequency of 0.5 Hz to the finger for 30 min. In contrast to spike-triggered microstimulation, persistent tactile stimulation produced a small but significant reduction in touch-induced firing rates. Similar results have been reported using voltage-sensitive dye imaging during rat whisker adaptation experiments [62]. However, there were no significant differences found for the Fano factor or the information-based measures (Figure 7B). Since the neural mechanisms underlying habituation are still not well understood, they have not yet been incorporated into the simulation, and hence it could not reproduce these results.

In summary, spike-triggered microstimulation had a stronger effect on the network than random microstimulation, and tactile versus electrical stimulation produced very different effects on the network. Thus, different stimulation protocols can be used to either facilitate or depress S1 activity and synchrony in response to natural touch. While the model performed well in reproducing both quantitative and qualitative aspects of the experimental spike-triggered microstimulation results, it was less successful at replicating the (null) results from random microstimulation and persistent tactile stimulation, most likely as a result of its lack of global activity patterns and a habituation mechanism. Further research will be conducted in the future to determine if we can bridge these gaps.

## Discussion

We found that (1) microstimulation in area 1 of the primate somatosensory cortex can induce neural reorganization at the level of both individual units and the ensemble; (2) this reorganization is dependent on the microstimulation protocol; (3) this reorganization appears to subside within 30 min (data not shown); and (4) a simulated network model of the thalamocortical system can successfully reproduce both qualitative and quantitative aspects of the *in vivo* findings. We found that a closed-loop, spike-triggered microstimulation protocol induced plasticity in area 1 that elevated the firing rate. We further found that this spike-triggered microstimulation decreased the variability of the ensemble, and caused changes in the functional connectivity between units, as quantified by mutual information and multi-information. The reference units used to trigger the microstimulation showed larger increases in mutual information than non-reference units. However, none of these changes were observed using random microstimulation. In addition, persistent tactile stimulation had the effect of a slight decrease in firing rate. While we believe the cortex is the most likely location for the neuroplastic changes underlying these effects to have occurred, it is possible that thalamocortical plasticity was also involved; as we did not record from the thalamus in these experiments, we could not directly address these possibilities.

### The Influence of Microstimulation on Neural Network Plasticity

The mechanism by which microstimulation affects neural dynamics is still not fully understood [32], [63]–[66]. The effect of microstimulation depends on the stimulation protocol parameters and the specific neural substrate, as well as the context within which it is applied. Microstimulation over several days has been found to cause structural changes between the thalamus and the cortex [67]. In our experiments, 30 min of microstimulation caused functional changes, but these did not lead to long-term effects, at least as detectable via our neurophysiological measures. However, we did observe a trend towards increased mutual information and response amplitude across several days (data not shown). One possible molecular mechanism for the long-term changes associated with microstimulation is modulation of adenosine A1 receptors; Márquez-Ruiz et al. [68] found that blocking the activation of these receptors in rabbits prevented the LTD in somatosensory cortex that was otherwise seen following transcranial direct-current stimulation.

We hypothesized that spike-triggered microstimulation would change network properties via Hebbian synaptic plasticity, but that random microstimulation would not, given the oscillatory environment of the rest of the brain. The lack of statistically significant changes in mutual and multi-information following random microstimulation appear to support this hypothesis; the fact that the model did not show this difference further supports the hypothesis that large-scale effects are involved. We also hypothesized that the synaptic changes are mostly due to potentiation, as these would be expected to result in increased unit-pair mutual information; however, in some sessions, we observed decreases in mutual information, suggesting that synaptic depression may also play an important role. We found that potentiation was especially pronounced among unit pairs that included the spike-triggered reference unit, as opposed to those that did not.

Following microstimulation, roughly equal proportions of units either became responsive to tactile stimulation or became nonresponsive, despite the fact that the overall responsiveness increased. This may indicate that there are regulatory mechanisms built into the neural dynamics that lead to a stable number of units representing touch to a given skin location. However, since the simulation produced nearly identical results despite lacking any explicit regulatory mechanism, this “regulation” appears to be a simple emergent property of the network.

In summary, our results show that microstimulation has clear short-term effects on network organization and modulation in awake animals. Such microstimulation could potentially be used to improve sensory performance by increasing the responsiveness of units to tactile stimuli, by increasing the efficiency of information transfer in individuals with compromised somatosensory throughput.

### The Effect of Microstimulation on Neural Coding

It has traditionally been believed that sensory information is encoded by firing rates [69]. Later, population coding was widely found in sensory and motor areas [70]–[72]. These coding strategies are based on integration, and are thus robust against noise. The role of neural synchrony is less clear, but may play an important roles in cognition such as arousal, expectation, vigilance, attention, and learning [73]–[77]. Synchronized firing has also been observed in anesthetized animals [78], and therefore may be a generalized property of the nervous system. Our data suggest that both coding strategies might be used in S1, since we observed increases in both firing rate and in information shared between pairs of units following microstimulation. Further research is needed to determine if it is possible to decouple these two effects, and whether each has a distinct influence on perception.

It has also been hypothesized that temporal coding could enhance information flow along sensory pathways and thus mediate sensory processing of external stimuli [71], [77], [79]. Synchronous responses are more likely than asynchronous ones to undergo rapid processing. This may aid the animal during active sensing to recognize external objects and guide motor actions when fast integration of sensory information is needed. We found that microstimulation increased synchrony, as measured by mutual information. However, additional psychophysical tests need to be performed to determine whether this produces measurable changes in perception. Recent reports have shown that stimulation in the sensorimotor cortex can enhance the detection threshold in rats [21] and that motor performance can be improved by rTMS stimulation in M1 in Parkinson’s disease [24].

### Conclusion

The effects that microstimulation of the primary somatosensory cortex has on neural dynamics depend strongly on the stimulation procedure used. Stimulations that are anchored to the underlying dynamics of the region can cause neuroplasticity that increases the network’s response amplitude to incoming sensory inputs, decreases the ensemble variability, and increases the mutual information shared by units that make up the neural ensemble. These effects were reproduced using a computer model of the thalamocortical system, providing a convenient and flexible alternative to *in vivo* experimentation for exploring microstimulation protocols. We do not yet know whether the network reorganization we observed following microstimulation will affect perception and behavior; this topic will be explored in future work.
