# Correction: Cortical Plasticity Induced by Spike-Triggered Microstimulation in Primate Somatosensory Cortex

**DOI:** 10.1371/annotation/a697686c-a062-4520-a9df-d5d0bbb42c7d

**Published:** 2013-03-08

**Authors:** Weiguo Song, Cliff C. Kerr, William W. Lytton, Joseph T. Francis

There were errors in the Author Contributions section. The correct contributions are: Conceived and designed the experiments: WS JF. Performed the experiments: WS. Analyzed the data: WS. Interpreted the data: JF. Designed the simulations: CK WL. Performed and analyzed the simulations: CK. Wrote the paper: WS CK JF.

