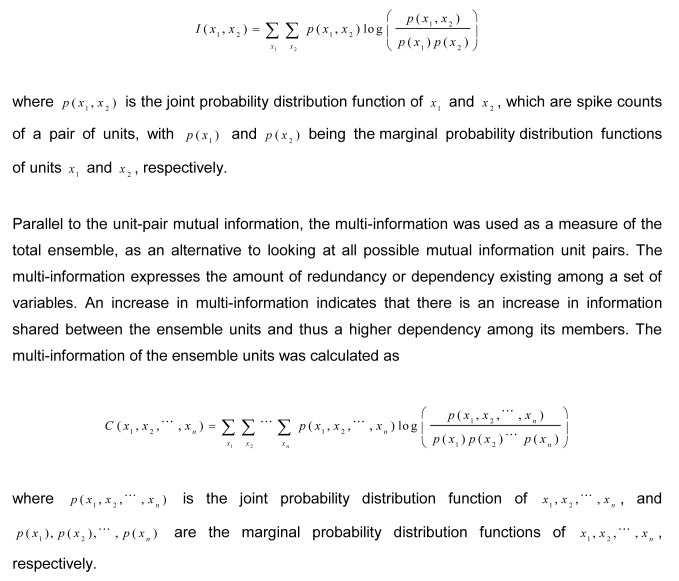# Correction: Cortical Plasticity Induced by Spike-Triggered Microstimulation in Primate Somatosensory Cortex

**DOI:** 10.1371/annotation/26c3d7a9-80dc-4719-bf61-968e5f322983

**Published:** 2013-03-13

**Authors:** Weiguo Song, Cliff C. Kerr, William W. Lytton, Joseph T. Francis

In the Materials and Methods section, there were errors in the equations in the section titled “Unit pair mutual information and multi-information.” Please view the complete, correct equations here: 

**Figure:**